# ID 319 - Avaliação do RAPID3 como Ferramenta de Triagem em Pacientes com Artrite Reumatoide no SUS

**DOI:** 10.5327/2237-9622.2025.v34s1.319

**Published:** 2025-11-25

**Authors:** Ilka Benedet Lineburger, Claiton Viegas Brenol

**Keywords:** artrite reumatoide, RAPID3; saúde digital, Avaliação de Tecnologias em Saúde, SUS, telemedicina, triagem de atividade de doença

## Abstract

**Introdução:**

A artrite reumatoide (AR) é uma doença inflamatória crônica que exige monitoramento contínuo da atividade da doença. O (RAPID3), no Questionário de Avaliação Multidimensional em Saúde (MDHAQ), tem sido utilizado como uma ferramenta simples de avaliação autorreportada para acompanhamento da atividade da AR. Este estudo tem como objetivo avaliar a sensibilidade e a especificidade do RAPID3 para identificar pacientes com atividade moderada/alta da doença no Sistema Único de Saúde (SUS), comparando seus resultados com o DAS28 (VHS) – padrão-ouro.

**Método:**

Foram incluídos 129 pacientes com diagnóstico de AR atendidos no Hospital de Clínicas de Porto Alegre (SUS), convidados a responderem o instrumento em formato eletrônico, previamente a suas consultas reumatológicas eletivas. O estudo teve aprovação do Comitê de Ética em Pesquisa desse Hospital e todos os pacientes preencheram o Termo de Consentimento Livre e Esclarecido. A utilização do questionário neste estudo teve prévia autorização do autor do instrumento (Pincus .).

Perfil dos participantes:

– Idade média: 59 anos (± 13)
– Gênero: 83,7% mulheres (n=108)
– Etnia: 90,7% brancos (n=117)

Dados clínicos:

– Tempo de doença (mediana): 13 anos (IQR 7-23)
– Fator reumatoide ou anti-CCP positivo: 86,7%
– Uso de medicamentos:
  - DMARD sintético: 81,4% (n = 105)
  - DMARD biológico: 31% (n = 40)
  - corticoide: 34,1% (n = 44)

**Resultados:**

– Sensibilidade e especificidade do RAPID3 para identificar atividade moderada/alta:
  - sensibilidade: 98%
  - especificidade: 49%
  - valor preditivo negativo: 92%
  - probabilidade pós-teste negativa: 8%
  - curva ROC do RAPID3: AUC = 0,79 (IC 95% 0,71-0,88)
– Facilidade de uso do instrumento em formato eletrônico:
  - 90% dos pacientes relataram facilidade no preenchimento do questionário;
  - 93% consideraram o RAPID3 uma boa ferramenta de avaliação;
  - a escolaridade não teve influência significativa no preenchimento (p=0,128).

**Conclusão:**

Discussão: o RAPID3 demonstrou ser uma ferramenta eficaz para a triagem de atividade de doença em pacientes com AR no SUS, especialmente devido à sua alta sensibilidade e ao valor preditivo negativo, comparável ao DAS28. Além disso, o questionário apresentou aplicabilidade prática tanto no formato eletrônico quanto em papel, com alta aceitação entre os pacientes. A correlação com desfechos clínicos e a facilidade de uso sugerem que o RAPID3 pode ser útil em contextos de atendimento remoto, como telemedicina, promovendo uma abordagem compartilhada entre médico e paciente. A utilização de questionários autorreportados pelo paciente (PROs) facilita a dinâmica compartilhada entre médico e paciente com impacto direto na tomada de decisões clínicas. Ao considerarmos a integralidade da avaliação pelo MDHAQ, diferentes estudos demonstraram sua capacidade de monitorar e contribuir positivamente na decisão compartilhada em diferentes patologias reumatológicas. Além disso, o MDHAQ permite a extração de escores pertinentes para identificação de fibromialgia, ansiedade e depressão sobreposta nessa população de pacientes.

**Conclusão:**

o RAPID3 mostrou-se confiável na triagem de atividade de doença em pacientes com AR, validado frente ao DAS28, tanto no formato papel quanto eletrônico. Sua utilização pode servir para melhorar o monitoramento de pacientes no SUS, especialmente em ambientes de cuidado remoto, reforçando sua importância na gestão de doenças crônicas.

